# Bilateral carotid artery geometry using magnetic resonance angiography: a 10-year longitudinal single center study

**DOI:** 10.1038/s41598-022-09062-7

**Published:** 2022-03-23

**Authors:** Woocheol Kwon, Yeryung Kim, Jisu Kim, Junsik Jo, Seongju Jeon, Ui Yun Lee, Hyo Sung Kwak

**Affiliations:** 1 Jeonbuk National University Medical School, Jeon-ju, 54907 Korea; 2 Division of Mechanical Design Engineering, College of Engineering, Jeonbuk National University, Jeon-ju, 54896 Korea; 3 Department of Radiology and Research, Institute of Clinical Medicine of Jeonbuk National University, Biomedical Research Institute, Jeonbuk National University Hospital, 20, Geonji-ro, Deokjin-gu, Jeonju-si, Jeollabuk-do 54907 Republic of Korea

**Keywords:** Diseases, Medical research, Risk factors

## Abstract

Aging and atherosclerotic progression can lead to geometric changes in the carotid arteries. We conducted a longitudinal study to investigate geometric changes in the bilateral carotid arteries. We conducted a retrospective study of 177 subjects who underwent carotid contrast-enhanced magnetic resonance angiography (MRA) at our clinic at baseline and 10 years after the procedure. Semi-automated methods were used to segment the bilateral carotid arteries to obtain carotid artery geometric measurements. The mean age for the total population after 10 years was 70.7 ± 10.6 years (male, 40.1%). The mean time interval between baseline and after 10 years for all subjects was 130.2 ± 8.1 months. The bilateral bifurcation angle, the diameters for both common carotid arteries (CCAs), and areas of both CCAs significantly increased (*p* < 0.001) over a 10-year period. The maximum diameter and internal carotid artery area did not significantly change. The bifurcation angle of the right carotid artery was significantly increased compared to the left carotid artery. However, the diameter and area of the CCA of the left carotid artery was significantly increased compared to the right carotid artery. The bifurcation angle, diameter and area of both CCAs significantly increased over a decade. The change in the bifurcation angle over a 10-year period was predominant in the right carotid artery and the change of the area and diameter of the CCA was dominant in the left carotid artery.

## Introduction

Carotid atherosclerosis is an important prognostic indicator due to its close relationship to the incidence of stroke. An increasing severity of stenosis is related to a higher risk of stroke^1,2^. Arterial remodeling occurs as atherosclerosis progresses^3^. During this process, the artery initially enlarges as plaque accumulates, to ensure an adequate blood flow. This enlargement of the artery is known as expansive remodeling. The geometry of the carotid artery influence hemodynamics, so it has been hypothesized as a risk factor for the initiation of atherosclerosis^4–10^. Recent studies have reported that vessel volume, diameter and the bifurcation angle of the carotid artery and internal carotid artery (ICA) increased with age^11^. Studies have shown that expansive remodeling of the carotid artery correlates with the risk of ischemic stroke^12^.

According to a population-based study, atherosclerotic plaque in the carotid artery is not necessarily characterized by bilateral symmetry^13^. Further, plaque in the left carotid artery is more vulnerable than in the right artery; however, the study did not compare the geometry of carotid artery. Therefore, to better understand the clinical implications, we compared both geometrical sides of the carotid artery. Furthermore, to date there are a limited number of longitudinal studies that have assessed the carotid artery geometry^14^. Most studies of carotid geometry compared young patients with older healthy subjects^9^. Accordingly, existing studies regarding age-related carotid artery geometry is insufficient. Therefore, we designed a longitudinal study to analyze both the right and left carotid artery diameter and bifurcation angle in each patient and changes that occurred over a decade.

The purpose of our study was to use a longitudinal study method to investigate geometric changes in the carotid artery. We hypothesized that the diameter of the common carotid artery (CCA), ICA, external carotid artery (ECA), and the bifurcation angle would increase over a 10-year period; additionally, we expected that expansive remodeling of the carotid artery would differ between the two sides.

## Materials and methods

This study was approved by the Institutional Review Board (IRB) of our institute, and the requirement for informed consent was waived by the IRB due to the retrospective design and imaging analysis approach of the study.

### Study population

The study population was taken from the dataset of the Jeonbuk National University Hospital. This present study was designed for the retrospective research that included subjects who underwent magnetic resonance angiography (MRA) at least twice during 10 years from 2008 to 2020, with 300 patients that responded to the baseline surveys. This paper uses a dataset from 263 subjects, who underwent MRA at baseline and after about 10 years after the longitudinal follow-up. The MRAs were performed to assess various clinical issues. The inclusion criteria were as follows: contrast-enhanced MRA examination at baseline and after 10 years with the same MR machine, no interval change in carotid stenosis without plaque, and acceptable imaging quality. Clinical data for the subjects, including basic demographics and risk factors for atherosclerosis, namely diabetes, hypertension, dyslipidemia, current smoking, and history of coronary disease, were also recorded.

### MR imaging protocol

MR imaging of the brain and carotid angiography was performed with a 3.0 T scanner (Verio; Siemens, Erlange, Germany) at baseline and after about 10 years. Contrast-enhanced carotid MRA was performed in the coronal plane using a T1-wighted spoiled gradient-echo sequence. The initial parameters of this study were as follows: ratio of repetition time (TR) to time to echo (TE) = 3.8/1.5 ms, flip angle = 30°, slice thickness = 0.90 mm, matrix size = 384 × 180, field of view = 183 × 300 mm, echo train length = 1, average number = 1, and acquisition time = 90 s. The parameters after 10 years were as follows: TR/TE = 3.3/1.2 ms, flip angles = 21°, slice thickness = 1.0 mm, gapless, matrix size = 448 × 280, field of view = 281 × 360 mm, echo train length = 1, number of average = 1, and acquisition time = 90 s.

They assessed image quality by consensus as visualization of vessel wall and degree of venous contamination. Poor imagings with ill-defined margin of carotid artery or no separation between carotid artery and jugular vein due to venous contamination were excluded from the final analysis. Disagreements regarding image quality were resolved by consensus. 

### Imaging reconstruction

Contrast-enhanced carotid MRA of the carotid artery was performed for all subjects. Both carotid arteries were segmented with semi-automatic software (MIMICS; Materialise, Leuven, Belgium) using coronal source data.

### Carotid artery geometry measurements

**Figure 1:**
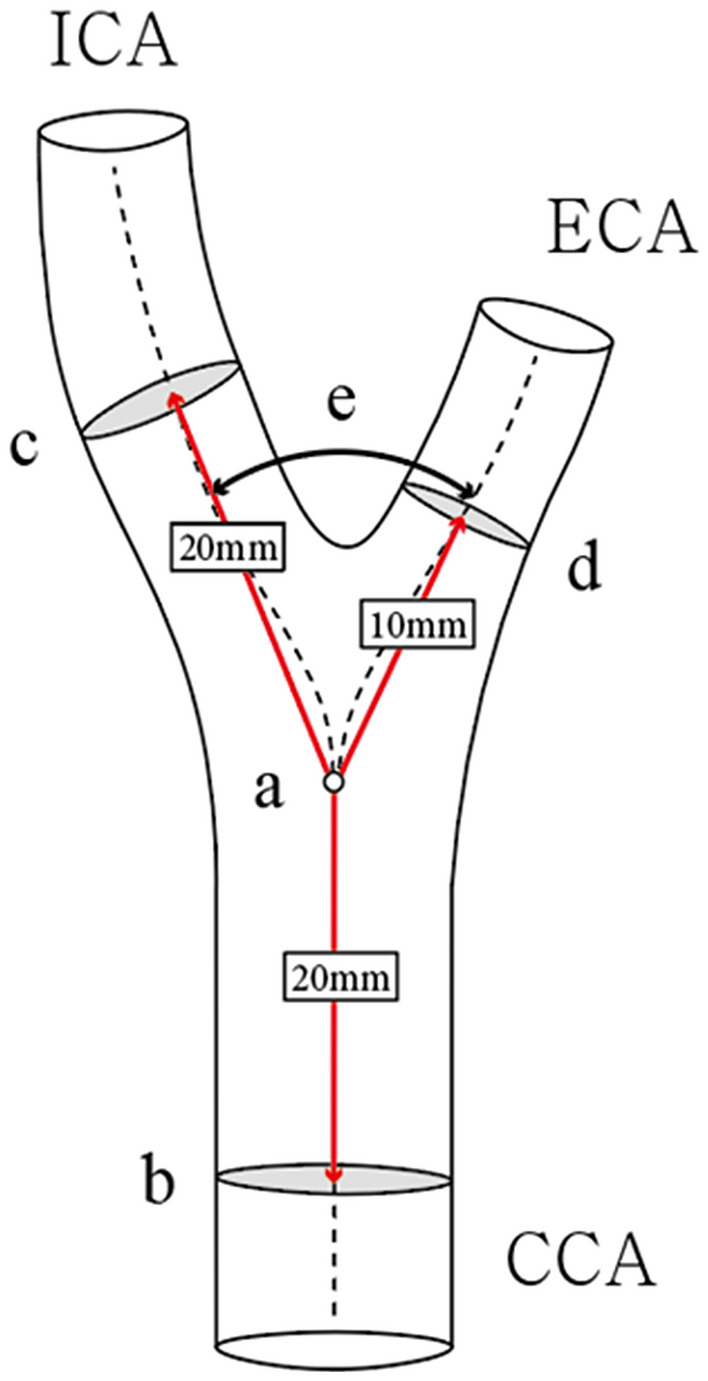
Geometric parameters of the carotid artery.

The geometric measurements for both carotid arteries were initially proposed by Thomas et al.^9^. Figure 1 presents the imaging reconstruction process and factors for measurement. Using MIMICS software, the arterial central lines were generated individually from the CCA to the ECA and to the ICA. The carotid bifurcation angle using central lines was defined as the angle between the ECA projections and ICA vectors at the carotid artery bifurcation. The diameter and area of the ICA and CCA were measured at a distance of 2 cm from the carotid artery bifurcation. The ECA diameter was measured at a distance of 1 cm from the bifurcation of carotid artery because of the influence of arterial branches. Additionally, the maximum diameter and area of the CCA, including the carotid bulb, were measured. The minimum diameter and area of the ICA were also measured.

### Statistical analysis

Statistical analysis was performed using SPSS version 24.0 (IBM Corp., Armonk, NY). The normality test was not conducted due to the large size of sample numbers. Geometrical changes of each carotid artery between baseline and after 10 years on MRA were assessed by paired T-tests. Comparison of geometrical parameter of both carotid arteries was performed by paired T-tests. All levels of significance were determined with a *p* value of < 0.05.

### Institutional Review Board Statement

This study was conducted according to the guidelines of the Declaration of Helsinki, and approved by the Institutional Review Board of Jeonbuk National Hospital (JUH 2019-10-041).

### Informed consent

Our institutional review board approved this prospective study, and patient informed consent was received before the MR examination.

## Results

### Subjects

**Figure 2:**
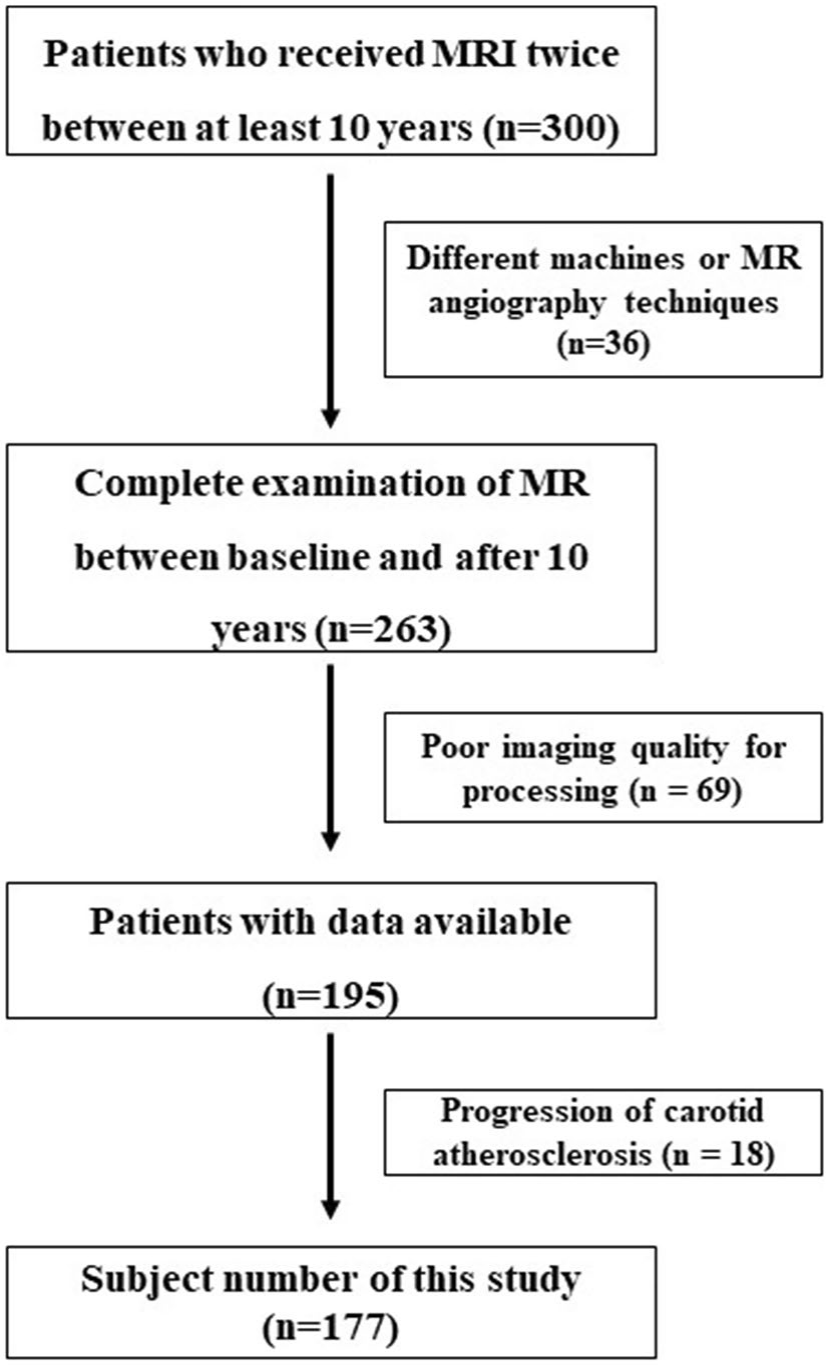
Subject selection.

We selected three hundred patients who underwent carotid MR angiography during a defined period based on a dataset from our hospital. Of these patients, 36 were excluded from this study due to the use of different MR machines or MRA techniques, and 69 were excluded because of poor imaging quality such as flow artifact or venous contamination for imaging processing using MIMICS. Further, 18 patients were excluded due to carotid stenosis with plaque that was identified during the follow-up period. In total, 177 patients (mean age after 10 years, 70.7 years; 106 females) were included this study (Fig. 2). The basic demographic data of subjects in this study are shown in Table 1. The mean time interval between two MRA examinations was 130.0 ± 9.2 months.

**Table 1:** Subject demographic data (after 10 years).

	Basic data
Mean age, years	70.7 ± 10.6
Female, n (%)	106 (59.9)
Hyperlipidemia, n (%)	31 (17.5)
Smoking, n (%)	28 (15.8)
Diabetes, n (%)	60 (33.9)
Hypertension, n (%)	113 (63.8)
Previous stoke event, n (%)	75 (42.4)
Cardiac problem, n (%)	32 (18.1)
Mean follow-up period, months	130.0 ± 9.2

### Geometric changes in each carotid artery

The geometric changes in each carotid artery and statistical analysis for the parameters are summarized in Tables 2 and 3. Bilateral bifurcation angles significantly increased over a 10-year period (*p* < 0.001; right: 47.46 ± 1.26° for baseline vs. 49.9 ± 1.25°, left: 43.98 ± 1.14° for baseline vs. 45.96 ± 1.17°). The diameters and areas of bilateral CCAs significantly increased over a 10-year period (*p* < 0.001). However, the diameters and areas of bilateral ICAs at the reference site decreased during the follow-up period. Especially, the diameters and areas in the left ICA at the reference site were significantly decreased (*p* < 0.05), and the diameters and areas of the bilateral ECAs at the reference site were significantly decreased during follow-up period (*p* < 0.05).

**Table 2:** Geometric changes in the right carotid artery from baseline and after 10 years of follow-up.

Geometrical parameters	Initial	After 10 years	Change over 10 years	Rate of Changes (%)	*p* value
Bifurcation angle	47.46 ± 1.26	49.9 ± 1.25	2.44 ± 0.53	4.35 ± 1.05	< 0.001
CCA diameter	6.69 ± 0.08	6.98 ± 0.08	0.29 ± 0.06	3.31 ± 0.96	< 0.001
CCA area	35.37 ± 0.8	39.06 ± 0.9	3.7 ± 0.69	6.05 ± 1.94	< 0.001
ICA diameter of reference	5.45 ± 0.07	5.35 ± 0.08	− 0.1 ± 0.06	− 3.88 ± 1.37	0.101
ICA area of reference	23.43 ± 0.68	23.17 ± 0.75	− 0.26 ± 0.51	− 9.49 ± 3.68	0.606
CCA maximum diameter	8.11 ± 0.1	8.12 ± 0.11	0.01 ± 0.07	− 1.14 ± 1.12	0.913
CCA maximum area	52.34 ± 1.37	53.18 ± 1.4	0.85 ± 0.85	− 3.13 ± 2.74	0.322
ICA minimum diameter	5.41 ± 0.07	5.31 ± 0.08	− 0.11 ± 0.06	− 4.08 ± 1.4	0.084
ICA minimum area	23.12 ± 0.67	23.33 ± 0.83	0.21 ± 0.59	− 8.83 ± 3.58	0.716
ECA diameter	5.01 ± 0.07	4.84 ± 0.07	− 0.18 ± 0.05	− 4.61 ± 1.19	0.001
ECA area	19.72 ± 0.57	18.77 ± 0.53	− 0.95 ± 0.41	− 8.68 ± 2.48	0.023

*CCA* common carotid artery, *ICA* internal carotid artery, *ECA* external carotid artery.

**Table 3:** Geometric changes in the left carotid artery from baseline and after 10 years of follow-up.

Geometrical parameters	Initial	After 10 years	Change for 10 years	Rate of Changes (%)	*p* value
Bifurcation angle	43.98 ± 1.14	45.96 ± 1.17	1.98 ± 0.55	3.21 ± 1.19	< 0.001
CCA diameter	6.81 ± 0.08	7.25 ± 0.08	0.44 ± 0.06	5.76 ± 0.81	< 0.001
CCA area	36.84 ± 0.9	41.58 ± 0.94	4.74 ± 0.69	6.44 ± 4.43	< 0.001
ICA diameter of reference	5.45 ± 0.07	5.30 ± 0.07	− 0.15 ± 0.05	− 3.78 ± 1.04	0.006
ICA area of reference	23.24 ± 0.58	22.38 ± 0.59	− 0.85 ± 0.43	− 7.97 ± 2.18	0.05
CCA maximum diameter	7.95 ± 0.1	7.97 ± 0.09	0.02 ± 0.06	− 0.17 ± 0.88	0.747
CCA maximum area	49.92 ± 1.29	50.79 ± 1.22	0.88 ± 0.76	0.51 ± 1.6	0.248
ICA minimum diameter	5.42 ± 0.07	5.27 ± 0.07	− 0.14 ± 0.05	− 3.75 ± 1.05	0.008
ICA minimum area	23.0 ± 0.57	22.28 ± 0.59	− 0.73 ± 0.46	− 7.62 ± 2.22	0.114
ECA diameter	5.09 ± 0.08	4.92 ± 0.07	− 0.18 ± 0.06	− 5.85 ± 1.96	0.001
ECA area	20.3 ± 0.6	19.77 ± 0.6	− 0.61 ± 0.45	− 7.45 ± 2.48	0.179

*CCA* common carotid artery, *ICA* internal carotid artery, *ECA* external carotid artery.

### Geometrical changes in bilateral carotid arteries over 10 years

**Figure 3:**
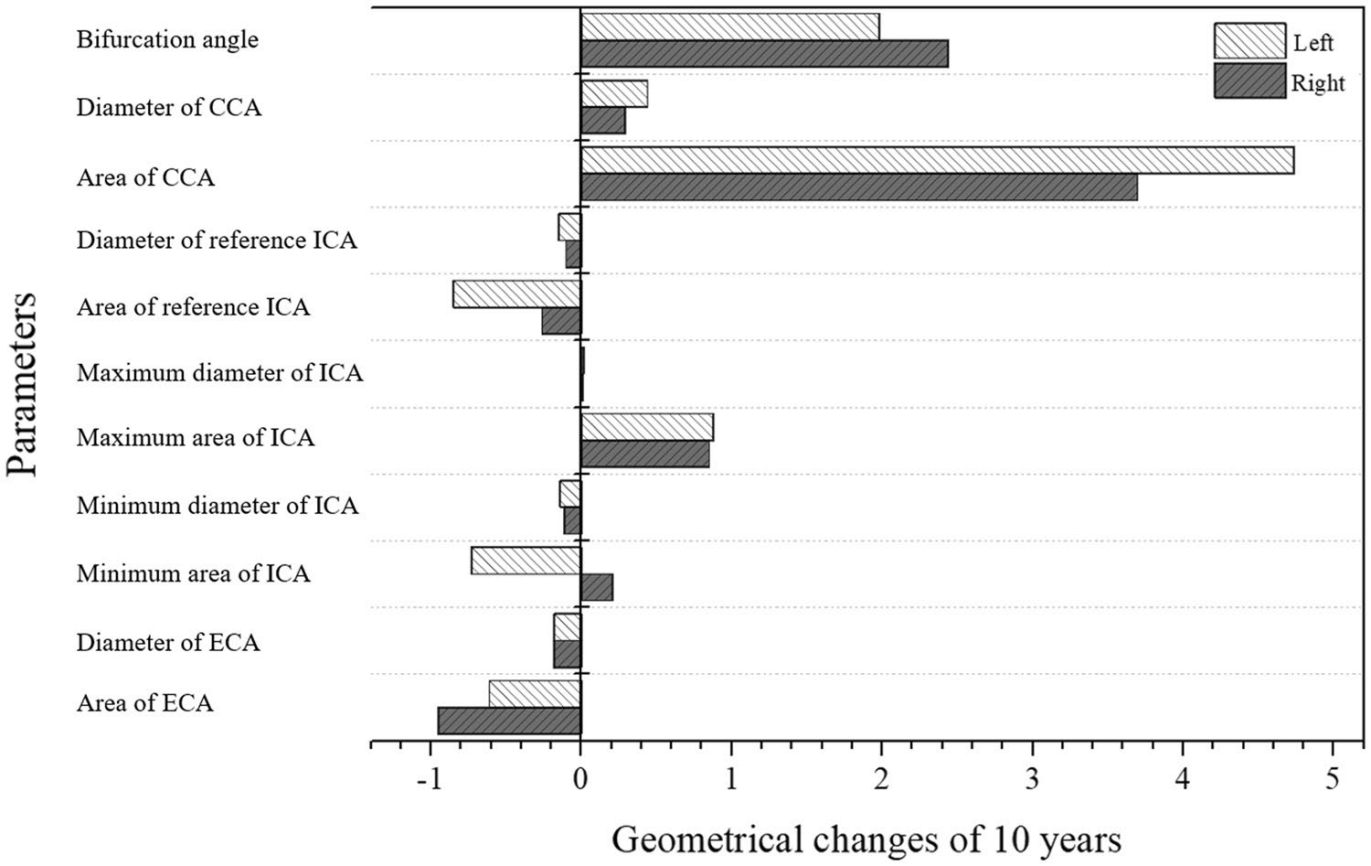
Geometric changes in bilateral carotid arteries over 10 years.

**Figure 4:**
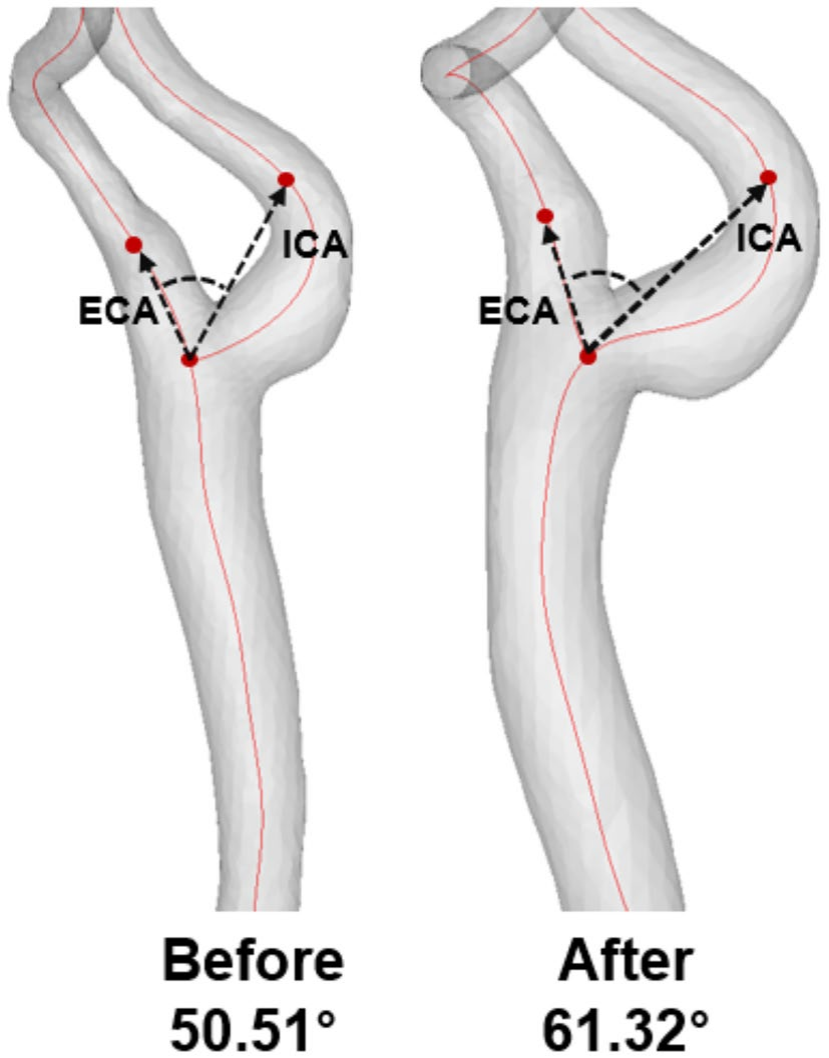
The representative case (left side) of geometric changes over 10 years.

Comparisons of geometrical changes in the bilateral carotid arteries over 10 years and associated statistical analysis are summarized in Table 4 and Fig. 3. The bifurcation angle of the right carotid artery was significantly increased compared to the left carotid artery (*p* < 0.001; 2.44 ± 0.53 vs. 1.98 ± 0.55) (Fig. 4). The diameter and area of the left CCA was significantly increased compared to the right CCA (*p* < 0.001), but the diameter and area of the left ICA at the reference site and the minimum diameter and area of the left ICA were significantly decreased compared to the right side (*p* < 0.001).

**Table 4:** Geometric changes in both carotid arteries after a 10-year follow-up period.

Geometrical changes over 10 years	Right	Left	*p* value
Bifurcation angle	2.44 ± 0.53	1.98 ± 0.55	< 0.001
CCA diameter	0.29 ± 0.06	0.44 ± 0.06	< 0.001
CCA area	3.7 ± 0.69	4.74 ± 0.69	< 0.001
ICA diameter	− 0.1 ± 0.06	− 0.15 ± 0.05	< 0.001
ICA area	− 0.26 ± 0.51	− 0.85 ± 0.43	< 0.001
CCA maximum diameter	0.01 ± 0.07	0.02 ± 0.06	< 0.001
CCA maximum area	0.85 ± 0.85	0.88 ± 0.76	0.726
ICA minimum diameter	− 0.11 ± 0.06	− 0.14 ± 0.05	< 0.001
ICA minimum area	0.21 ± 0.59	− 0.73 ± 0.46	< 0.001
ECA diameter	− 0.18 ± 0.05	− 0.18 ± 0.06	1.000
ECA area	− 0.95 ± 0.41	− 0.61 ± 0.45	< 0.001

*CCA* common carotid artery, *ICA* internal carotid artery, *ECA* external carotid artery.

## Discussion

In this study, we investigated geometric changes in the bilateral carotid arteries that occurred over the course of a decade and without carotid stenosis. We identified cases with follow-up over a 10-year period, and the bilateral bifurcation angle, diameters of both CCAs and areas of both CCAs all increased significantly. Kamenskiy et al.^15^ also demonstrated that bulb diameter and bifurcation angle increase in carotid arteries with increasing age. However, we found that the maximum diameter and area of the ICA did not significantly change. We also found that the diameters and areas of the bilateral ICAs at the reference site decreased during the follow-up period. This result is supported by the study by Thomas et al. which reported that old vessels exhibited greater bifurcation angles, as well as smaller ECA/CCA and ICA/CCA diameter ratios^9^. The results of this study indicated that the bifurcation angle of the right carotid artery was significantly increased compared to the left carotid artery; additionally, the diameter and area of the left CCA were significantly increased compared to the right CCA.

Arteries are frequently exposed to radial, circumferential, and longitudinal forces that are exerted on their wall or as shear stress on the endothelial surface^16^. With age, vascular smooth muscle cells undergo functional changes that alter the normal structure of the vessel wall, which progresses to atherosclerosis. Elastin fragmentation attributable to mechanical fatigue results in passive dilatation and stiffening of the regional aortic wall^17^. Kamenskiy et al.^15^ reported that the continuity and density of the circumferential and longitudinal elastin in the CCA and ICA decrease with age.

Variations in the geometry of the carotid bifurcation increase significantly with age or early atherosclerotic progression^8,9^. However, there are currently limited data that have assessed longitudinal impacts on carotid artery geometry^14^. These data typically come from studies that compare carotid geometry in young people with older healthy subjects rather than directly studying an individual person^9^. Further, carotid bifurcation geometry is significantly correlated with the incidence of stroke^18^. Therefore, measuring the ICA angle may help assess the risk of ischemic stroke and the ICA angle could be used as a possible risk factor for assessing ischemic stroke. The ICA angles were significantly larger in patients with history of a stroke than in controls^19^. Consequently, we hypothesize that the longitudinal study of carotid bifurcation geometry can help predict the risk of stroke.

The geometry of carotid arteries has been considered as an important potential risk factor for the early development of atherosclerosis because of its significant influence on local hemodynamics^20^. Likewise, the bifurcation structure can cause various local hemodynamics in carotid segments and therefore may significantly influence the difference and changes in segment atherosclerosis. A previous study reported that the increase of the carotid bifurcation angle was related to carotid remodeling because of aging. With normal aging, the bifurcation angle gradually widens as the arteries dilate and become more tortuous. The carotid bifurcation angle became broader with age and increased by 10° over each decade of life^14,15^. In our study, the bifurcation angle of the bilateral carotid arteries significantly increased over 10 years. Additionally, the bifurcation angle of the right carotid artery significantly increased compared to the left carotid artery. However, the degree of increase of the bifurcation angle was approximately 2° over the 10-year follow-up period.

Atherosclerotic plaques in the left carotid artery were more vulnerable to conditions such as intraplaque hemorrhage and thin fibrous caps, compared with the right^13,21^. Our study did not identify geometrical changes in both carotid arteries over the 10-years of follow-up. The bifurcation angle of the right carotid artery significantly increased, but the diameter and area of the left CCA significantly increased. The diameter and area of the left ICA at the reference site and the minimum diameter and area of left the ICA were significantly decreased compared to the right side (*p* < 0.001). Further, some studies have shown that the diameter and circumference of the ICA increase with age^11^, while other studies have not reported significant changes over 10 years^20^. In our study, the changes in the diameter and area of the ICA were different in the left and right sides. The area and diameter of the ICA decreased in the left side, but significant changes were not noted in the right side. Differences in these changes between both carotid arteries could be attributable to geometric factors, such as the bifurcation angle, the configuration of the left carotid artery with the aortic arch or because of the direct connection of the left carotid artery to the aortic arch, as opposed to the right carotid artery that arises from the brachiocephalic artery^8,13,22^. Vessel anatomy influences hemodynamic forces, and as such, the left carotid artery may be exposed to higher arterial pressures^8,13,22^. Therefore, the right carotid artery with an increased bifurcation angle can experience hemodynamic forces that cause changes in the carotid bulb, and the left carotid artery, with the increased area and diameter of the CCA, can experience the hemodynamic forces that are changed in the CCA.

This study had some limitations. First, it was a retrospective study, which includes the limitation of the inherent possibility of selection bias. Second, because the carotid MRAs were performed for clinical indications, the included subjects were more likely than the general population to have vascular risks. However, the inclusion criteria of subjects with only normal VBA systems may have helped diminish this effect. Third, during the 10 years of follow-up, different parameters of the carotid MRA sequence were used, due to advances in techniques. However, we analyzed the data set based on examinations performed with one MR machine. Fourth, MRA was used for geometrical analysis of carotid artery instead of using CT angiography. Although MRA often had poor imaging quality due to flow artifact or venous contamination, we utilized contrast-enhanced MRA which showed relatively excellent visualization of vessel lumen. Moreover, in our hospital, MR examination is more common examination compared to CT angiography due to radiation dose and additional information of brain. Finally, because our study was not intended to be an epidemiological study, our patients do not necessarily represent characteristics of the general population.

## Conclusion

Our longitudinal study was based on changes in individuals during a long-term follow-up and showed that the bifurcation angle, diameter and area of both CCAs significantly increased over a decade of life. Additionally, geometrical change of both carotid arteries over 10 years was not symmetrical. Changes in the bifurcation angle over a 10-year period were dominant in the right carotid artery and changes in the area and diameter of the CCA were dominant in the left carotid artery. Further studies are required to evaluate the association between geometrical changes and atherosclerotic risk factors.
